# What Lies Beneath: A Development-Oriented Auditing Approach to Understand Organizations Beyond the Surface of Hard-Control

**DOI:** 10.1177/00332941241283199

**Published:** 2024-10-13

**Authors:** Jorrick Beckers, José Hermanussen, Ronald Stevens, Ingrid van der Pluym

**Affiliations:** Department of Social Learning, 10198 Open University of the Netherlands, Heerlen, The Netherlands; 385045 Expertisecentrum Beroepsonderwij's, Hertogenbosch, The Netherlands; Het Zijlstra Center, 1190 Vrije Universiteit, Amsterdam, The Netherlands; Kwaliteitsnetwerk mbo, Stichting Kwaliteitsnetwerk Mbo, Nijmegen, The Netherlands

**Keywords:** Organizational culture, organizational psychology, employment psychology & marketing, motivation, employment psychology & marketing, leadership, organizational psychology, employment psychology & marketing, vocational education, soft-control, development-oriented audit

## Abstract

Auditing procedures aim to improve educational quality in vocational education and training. Auditing approaches often focus on checking for compliance to rules and standards. Through dialogue, development-oriented audits are thought to inspire soft-control, a form of control that aims to address what is beneath the surface of mere compliance. These kinds of audits offer opportunities to demonstrate ethical leadership as part of an ethical culture. It is expected that an ethical culture variables positively influence working climate variables and ultimately intrinsic motivation. In this study, conducted as part of the (Hermanussen et al., 2022) study, we employed structural equation modeling to test if model behavior and sanctionability positively influenced perceived autonomy, perceived relatedness, trust, and self-efficacy, and ultimately intrinsic motivation. The study was conducted at eight different Dutch secondary vocational education and training organizations including 1223 participants. Results demonstrate a good model fit, χ2 = .12, df = 3, *p* = .989, GFI = 1.00, AFGI = 1.00, and RMSEA <.001. All presumed effects were significant. The strongest effects include model behavior on autonomy (.49) and autonomy on intrinsic motivation (.45). Future research should employ designs and analyses that are able to account for a multilevel structure in educational organizations.

## Introduction

Auditing refers to a procedure that is aimed at ensuring compliance to certain standards and values of a particular framework within organizations (Hayne & Salterio, 2014). While originating from corporate environments, audits are now widely applied within vocational education and training (VET) where they are used in a similar fashion. While audits are still used to check for adherence to norms, standards, and rules (both internally and externally), they are also used reveal possible risks in time for organizations to respond to or adapt to (Anthony & Govindarajan, 2001). Furthermore, audits in VET generally also ultimately aim to improve educational quality. However, this double aim (i.e., a quality control aim and an educational improvement aim) has not yet come to full fruition. Research indicates that employees in Dutch VET institutions primarily notice controlling aspects of institutional audits, while intended learning effects are often a lot less felt (Hermanussen & Brouwer, 2016).

Indeed, institutional audits tend to focus on quantifiable, measurable aspects of organizational performance (e.g., number of graduates and teacher evaluation scores), these are so-called ‘hard controls’ (Chtioui & Thiéry-Dubuisson, 2011) that are aimed at accountability. These kinds of audits tend to capture accountability aspects of organizational performance adequately. However, institutional audits with an accountability focus mostly include auditing teams that assume the role of inspectors looking for anomalies or deviations from the norm. This role, in favor of that of a critical friend, can lead to perceived lack of relevance among auditees and in the long run fail to lead to meaningful improvements in educational practice (Stevens, 2012). Actual improvements in educational practice are expected to occur by strengthening the prevailing quality culture (Den Boer & Frietman, 2014), a culture in which members of an organization motivate one and another to work continuously and sustainably towards improving educational quality.

Research suggests that to impact quality culture, auditing approaches need to go beyond steering on hard-control only, but rather also include soft-control in the process (e.g., Lenka et al., 2010). Soft-control refers to impacting emerging aspects of organizational quality that lie beneath the surface of the easily tangible. These aspects include but are not limited to trust and self-efficacy and are generally ignored by audits with a focus on hard-control. Organizations can enforce soft-control to impact emerging aspects positively, if the managerial level instils an ethical culture (Vink & Kaptein, 2008). For example, soft-control can be deployed if the managerial level leads by example (i.e., shows model behaviour) and promotes a culture in which norms and values are followed. Soft-control is also high if the managerial level shows that adhering to rules is rewarded, while acting in discordance with the rules is met with consequences (i.e., is sanctioned Venne et al., 2014).

The current study aims to determine if impacting soft-control as well as hard-control through auditing approaches is an avenue worth pursuing. We want to establish whether soft-control variables impact important aspects of organizational performance that lay underneath the surface of what is generally targeted by hard-control. Specifically, we aim to do so by unravelling relations between ethical culture variables (as measured by role model behavior and sanctionability), working climate variables (as measured by perceived autonomy, perceived relatedness, and self-efficacy) and intrinsic motivation. This study is part of another larger study (Hermanussen et al., 2022).

This study also adds to the existing literature broader understanding of the theoretical mechanisms of intrinsically motivating employees in an organizational context and aims to provide insight into which dials can be turned to support intrinsic motivation among employees.

## Theoretical Framework

### Audits

Organizations view audits as the ‘third line of defence’ in the Three Lines of Defence model (IIA, 2020), which is a model aimed at risk management and governance. While audits are the final line of defence, the first line includes risk management at a front office level (e.g., prudence among loan officers in case of banks), while the second line includes risk management at the back-office level, where supportive roles such as human relations and compliance are situated. Audits have made their way into the educational sector during the period of New Public Management, a period in which management techniques from the private sector steadily started to transition towards the public sector. Generally, audits aim to determine any substantial risks organizations face, whether they comply with, rules, norms, standards, and laws, and what points of improvement might be, based on a relevant frame of reference. Mostly, audits assess all the previous from a perspective that prioritizes control.

Following Stevens (2012) we discern two perspectives in assessing quality of public organizations (i.e., these include VET institutes), namely the control perspective and the developmental perspective. In the control perspective the driving notion is that performance can be measured and recorded based on carefully constructed indicators. This perspective prioritizes determining whether organizations are doing what they are supposed to. It is concerned with ascertaining all the details, over obtaining the big picture. In contrast, the developmental perspective views an organization organically, constantly adapting to, and interacting with its societal environment (Homan, 2005). As such, this perspective propagates that organizations use reflection and dialogue among others to adapt to changing demands, rather than trying to exert control over dynamic circumstances.

Arguably, auditing from a control perspective (i.e., a traditional audit) can be very effective when used on suitable dilemmas. However, using audits from a control perspective to effectuate sustainable improvements in educational practice might be problematic for three reasons. Firstly, educational dilemmas have grown more complex over the years, which may require audits to assume a more holistic approach uniting viewpoints from all relevant stakeholders (Nuijten et al., 2015). Secondly, auditing should help the participants involved to assume responsibility over their core dilemmas. However, traditional auditing seems to fail in creating a sense of urgency or responsibility over what was discussed among participants (Weick & Sutcliffe, 2011). Thirdly, auditing in the control perspective often neglects to impact organizational quality culture. It fails to address what is under the surface of procedures and rules. A different type of audit is needed to address shortcomings of traditional audits when it comes addressing dilemmas in the complex reality of educational practice in VET.

### Audits and Soft-Control

In the development-oriented perspective to auditing, dialogue is an important tool to enact soft-control; dialogue across hierarchical layers of an organization helps to make sense of more abstract concepts beyond the typical key performance indicators. In dialogue, all organizational members work together to create a quality culture as a foundation of constant improvement to educational practice. To emphasize the different focus of a developmental audit, sometimes different names such as ‘institutional dialogue’ are used. Ethical leadership is an important first building block of quality culture. Kaptein (2008) developed the ‘Corporate Ethical Virtue Model’ (CEV), distinguishing several different leadership virtues that inspire ethical behavior among employees. Two important virtues are congruence of management and sanctionability. Congruence of management inspires ethical behavior by showing that management itself acts in accordance with the norms; management acts a role model. Sanctionability stimulates ethical behavior by showing that unethical behavior will be punished, while ethical conduct will be rewarded.

Ethical leadership attempts to inspire employees to engage in ethical conduct and is also important in creating a sound working climate. We distinguish four important variables at the level of the working climate, which include (perceived) autonomy, (perceived) relatedness, trust, and self-efficacy. Perceived autonomy and perceived relatedness are part of self-determination theory (SDT; Deci & Ryan, 2012). SDT is a macro theory of human motivation, development, and wellness (Deci & Ryan, 2008). The theory posits that human beings have a natural tendency towards intrinsic motivation, yet their innate psychological needs for autonomy, relatedness, and competency need to be satisfied. In this theory motivation is understood in terms of quantity (i.e., amount of motivation) and quality (i.e., type of motivation). Importantly, SDT differentiates itself from other contemporary motivation theories by focusing on both instead of just motivation quantity, arguing that while it is problematic to have a lack of motivation, research shows that motivation quality is just as important as motivation quantity, if not more so (Vansteenkiste & Soenens, 2023). Regarding motivation quality, broadly speaking two types of motivation are distinguished, being autonomous motivation and controlled motivation. Autonomous motivation refers to subtypes of motivation that are more or less internally regulated, whereas controlled motivation refers to types of motivation that are more or less externally motivated. Needs-supporting practices can stimulate development of autonomous forms of motivation (Ten Cate et al., 2011). While self-determination theory is a macro theory of human motivation, numerous studies have placed it in organizational contexts (e.g., Deci et al., 2017).

Organizational trust has been defined in different ways. It generally refers to an interpersonal construct within organizations that combines mutual respect and a feeling of being able to count on each other. There are also a great number of studies that illustrate an important role of trust in relationship to performance (e.g., Karatepe et al., 2019). Finally, we distinguish self-efficacy on the working climate level. Like the other working climate variables, self-efficacy is originally a psychological construct referring to one’s own beliefs about being able to complete tasks at desired levels of performance (Bandura, 1994). Like the other constructs, self-efficacy has been studied thoroughly in organizational context and been linked to various important outcomes such as organizational effectiveness (Bandura, 2009). At the motivational level we focus on intrinsic motivation which refers to motivational quality, rather than quantity, it is a state of voluntary engagement. In studies across a plethora of domains intrinsic motivation is both a sought-after antecedent as well as an outcome. Organizational research is no different (e.g., Lin, 2007).

### Hypothesis

**Figure 1.**
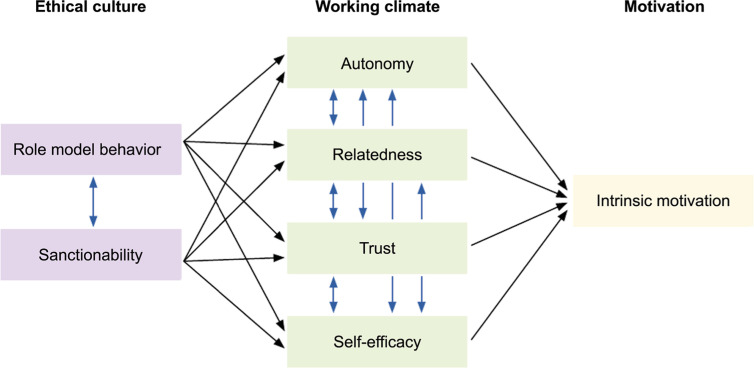
Conceptual model and hypothesized associations between ethical culture, working climate and motivation variables. Figure adapted from Hermanussen et al. (2022).

Based on the above we expect that by enacting soft-control through dialogue in development-oriented audits a chain of positive reactions can be initiated; when leaders act ethically (i.e., show role model behavior and apply sanctionability) there will be a positive impact on working climate and ultimately on intrinsic motivation. Specifically, we expect the following to occur. Sanctionability and role model behavior will influence one another, as Kaptein (2008) has previously established strong correlations between congruence of management with ethical expectations, congruence of supervisors with ethical expectations, and sanctionability. Research shows that ethical culture has been positively related to all the working climate variables. Pučėtaitė et al. (2015) for example demonstrate that ethical organizational culture can advance organizational trust, while Lumpkin and Achen (2018) point towards linkages between self-determination theory (of which perceived autonomy and perceived relatedness are a part) and ethical leadership. Finally, Wang et al. (2015) demonstrate that ethical leadership positively affects self-efficacy. Research also shows that the working climate variables are all related among each other. Firstly, self-determination theory stipulates linkages between perceived autonomy and perceived relatedness (Deci & Ryan, 2012). Furthermore, Wang and Li (2017) demonstrated that both perceived autonomy and perceived relatedness are related to trust. Dang and Chou (2019) demonstrated a positive relationship between trust and self-efficacy and Trépanier et al. (2012) established linkages between self-determination theory and self-efficacy. Finally, all the working climate variables have been found to be positively related to intrinsic motivation. Both, perceived autonomy and perceived relatedness are important conditions for intrinsic motivation (Ryan & Deci, 2000b). Joo et al. (2010) for example demonstrated a positive relationship between self-efficacy and intrinsic motivation, while Conchie (2013) established a positive relationship between trust and intrinsic motivation. In Figure 1 we specify a conceptual model and hypothesized associations between key variables in this study. The full hypothesis accompanying the conceptual model is as follows:

Hypothesis Ethical culture, concerning role model behavior and sanctionability will have a positive relationship with working climate variables, including perceived autonomy, perceived relatedness, trust, and self-efficacy, these variables are positively related to intrinsic motivation. Furthermore, we expect all ethical culture variables to be interrelated as well as working climate variables.

## Methods

### Context

This study was situated within a larger inquiry looking into the effects of a development-oriented audit approach on the development of quality culture and educational quality within secondary vocational education and training (SVET) organizations (Hermanussen et al., 2022). As part of the auditing approach soft-control data were also obtained. The audits were undertaken using a novel approach to auditing, based on the dialogue model (Stevens, 2018). The dialogue model entails a holistic, integral approach to auditing. While a detailed description of the model is beyond the scope of this article, we will describe its main features.

#### The Dialogue Model

The model is centred around five distinct capacities, which, as a whole form the backbone of a well-functioning organization. These include ‘Educational’, ‘Adaptive’, ‘Organizational’, ‘Professional’, and ‘Realization’ capacities. In developmental audits these five different capacities form areas of interest to help shape the dialogue. While these different capacities can be addressed separately, they cannot be approached as isolated parts. They should be approached as a holistic unit of interconnected parts. Please note that the content of the dialogue will be directed by questions VET-institutions formulate themselves. The different capacities help explore these questions in full. In the dialogue model there is a central focus on the educational capacity. This capacity refers to an organizational capability to offer solid, future-proof educational programs. This capacity is foundational to help students prepare for their vocations. Secondly, the adaptive capacity refers to an organizational capability to adapt to the demands of its surrounding environment. For example, a highly adaptive organization can adapt to the fact that we need professionals who know how to behave in a sustainable manner, even though that would imply changes within the organization. Thirdly, the organizational capacity refers to an organizational capability to run a VET institute in such a way that the organization can fulfil its goals and tasks. If we consider the previous sustainability example, a VET institute with a strong organizational capacity would have the structures and procedures in place to support the needed adaptations. Fourthly, the professional capacity refers to professional capability to realize high quality education at the individual-, team-, and the organizational level. Placed in the example, organizations that are professionally capable have individuals in place that recognize a need for learning new skills regarding sustainability and act accordingly. Finally, the realization capacity, refers to the capability to sharply determine to what the degree an organization has realized its goals. Again, in the sustainability example, a highly capable organization in terms of realization would be able to determine whether they have met their goals for sustainability.

#### Participants

In total eight different SVET organizations participated in this study. In the Netherlands SVET educates students on four different levels (SVET-1 through 4). These levels represent the various complexity levels of many vocations in the Netherlands (e.g., hairdresser, plumber, and welder). Typically, students enter SVET with a degree from junior vocational education when they are around 16 years old (but other routes are possible). If all goes well, they acquire in a degree within 2–4 years, depending on the level. An SVET-4-degree also grants access to higher vocational education. The SVET organizations included in our study were approached to take part right around the time they were due for a periodic internal audit either in 2018 or 2019. Participation occurred on a voluntary basis. In 2019 these organizations had between 2359 and 19,375 students and between 239 and 1751 FTE in staff. Details of each participating organization are described in Table 1.

**Table 1.** Details of Participating SVET Organizations.

Organization	Type	Number of students (2019)	Number of staff in FTE (2019)
1	RTC	16,424	1462
2	RTC	5200	380
3	RTC	2359	239
4	RTC	14,889	1223
5	RTC	12,199	1032
6	RTC	19,375	1751
7	RTC	13,372	1116
8	STS	5054	659

*Note.* RTC = Regional Training Centre for Vocational Education. STS = Specialized Trade School.

### Measurement Instruments

#### Soft-Controls Questionnaire

We constructed several scales to measure the variables of interest in our study. These variables included sanctionability, role model behavior, autonomy, relatedness, trust, self-efficacy, and intrinsic motivation. Construction of these scales (i.e., formation and selection of items) was based on relevant literature and existing questionnaires (Jansen In De Wal et al., 2016; Kaptein, 2008; Ryan & Deci, 2000a; Venne et al., 2014). Wording in the questionnaire was slightly adapted for every different layer of the SVET-organization (i.e., executive board, higher management, middle management, general staff, and teaching staff) to make sure questions were understood and meaningful. Feedback about the wording was gathered from one SVET institute (an institute from a pilot study not included in the current study) and used to improve the questionnaire.

Sanctionability was measured with four items (e.g., ‘In our organization we hold employees accountable if they harm educational quality’). Role model behavior was also measured with four items (e.g., ‘The behavior of my supervisor is congruent with the norms and values set by the board’). Autonomy was measured with four items (e.g., ‘I feel I can do my work in any way I see fit’). Relatedness was measured with seven items (e.g., ‘I feel a bond with my fellow team members’). Trust was measured with five items (e.g., ‘In my organization everyone treats one and other with respect’). Finally, self-efficacy was measured with six items (e.g., ‘I can complete complex tasks within my educational team’). The full questionnaire for the teaching staff layer is included in appendix 1.

### Procedure

#### Soft-Controls Questionnaire

The questionnaire was digitized using ‘Survalyzer’ and sent to participating SVET organizations at the beginning of their respective institutional audits. Questionnaires were distributed among employees of the SVET organizations for each layer of the organization separately (members of the board, higher management, middle management, supportive staff, and educational teams). Prospective participant contact information was obtained through central contact points at the participating organizations. Prospective participants subsequently received an invitation to fill out the questionnaire through their email addresses. Where necessary, a reminder was sent at two separate occasions. The first reminder was sent two weeks after the first invitation, a second reminder was sent two weeks after that. Additionally, the central contact points were asked to raise awareness about filling out the questionnaires as well as asking colleagues personally to participate. In total 2112 employees were approached of which 1223 filled out the questionnaire.

### Analysis

#### Factor and Reliability Analysis

To check whether these items constituted scales, we performed a Principal Component Analysis (PCA). For all the presumed constructs Keyser-Meyer-Olkin (KMO) values were acceptable to very good (values range from .67 to .83), indicating that PCA analyses could be performed (Strickland, 2003). Because we expected the various constructs to relate to one and other, we used oblique rotation (Oblimin). For almost all the constructs, a one-factor solution seemed to be most warranted, based on Eigenvalues and Scree plots. For the relatedness construct a two-factor solution would be advisable, based on the associated Eigenvalues (*>*1). However, the Scree plot suggested a one-factor solution (there was a sharp bend between the one-factor and two-factor solutions). Considering that the sample size is considerable (*N* = 1223) and the fact that theory supports a one factor solution over a two-factor solution, we chose to opt for the former. After PCA, we calculated the respective alphas to establish scale reliability. These alphas ranged from .66 to .89. Depending on the source, alphas from .70 and up are designated to be reliable. However, lower values may also be acceptable in the case of social/psychological constructs (Kline, 2000).

Table 2 presents the result of PCA based on one-factor solutions. The table includes Eigenvalues, percentage of variance explained by the factor and associated scale alphas.

**Table 2.** PCA Summary Based on One-Factor Solutions, Including Eigenvalues, Percentage of Variance Explained and Associated Alphas.

Scale	Eigenvalue	% of variance explained	*α*
Sanctionability	2.540	63.489	.81
Role model behavior	3.570	71.401	.90
Autonomy	2.021	50.520	.66
Trust	2.561	64.017	.82
Relatedness	3.462	49.461	.81
Self-efficacy	2.068	51.705	.67
Intrinsic motivation	2.730	68.244	.84

#### Path Analysis

To explore the relations depicted in the theoretical model in Figure 1 we employed ‘Structural Equation Modeling’ (SEM; Jöreskog & Sörbom, 1993) using LISREL 8.54. The structural parameters (i.e., standardized regression coefficients) were estimated using Maximum Likelihood Estimation (MLE). Based on the data, the LISREL program identifies which relations should be added or removed from the initial model to attain optimal model fit. Model fit is assessed using Chi-square and various fit indices including, Goodness of Fit Index (GFI), Adjusted Goodness of Fit Index (AGFI), and Root Mean Square of Approximation (RMSEA). A non-significant Chi-square closer to the degrees of freedom indicates a more optimal fit. An RMSEA *<*.08 indicates an acceptable fit while an RMSEA *<*.06 indicates a strong fit. GFI and AGFI need to be greater than .90 (Hu & Bentler, 1999).

### Research Ethics

This study was performed in accordance with the principles stated in the WMA Declaration of Helsinki. Our study was a survey of non-interventionist nature outside of the medical domain. These studies need not apply for ethical approval in the Netherlands. Participation in our study occurred on a voluntary basis. Participants were approached by local contact persons in every institute, who were also responsible for distribution of the questionnaire. Data were analysed anonymously (i.e., researchers worked with anonymised data).

## Results

### Descriptive Statistics

Table 3 displays descriptive statistics and correlations for all the variables used in this study. As expected, all variables significantly correlate to one and other. Correlations between variables range from moderate (e.g., between role-model behavior and self-efficacy; *r* = .32, *p* = .000) to large (e.g., between trust and relatedness, *r* = .72, *p* = .000).

**Table 3.** Descriptive Statistics and Correlations for all the Variables Used in This Study.

	M	SD	1	2	3	4	5	6	7
1. Sanctionability	3.34	.76	1.00						
2. Role model behavior	3.48	.83	.61^**^	1.00					
3. Autonomy	3.59	.66	.60^a^	.67^a^	1.00				
4. Trust	3.49	.80	.65^a^	.68^a^	.64^a^	1.00			
5. Relatedness	3.67	.63	.62^a^	.61^a^	.67^a^	.72^a^	1.00		
6. Self-efficacy	3.73	.61	.41^a^	.32^a^	.57^a^	.42^a^	.51^a^	1.00	
7. Intrinsic motivation	4.01	.69	.45^a^	.42^a^	.68^a^	.46^a^	.62^a^	.58^a^	1.00

*Note.* 1225 ≤ *N* ≤ 1233.

^a^Significant at the *p* < .01 level.

### Structural Equation Modelling

**Figure 2.**
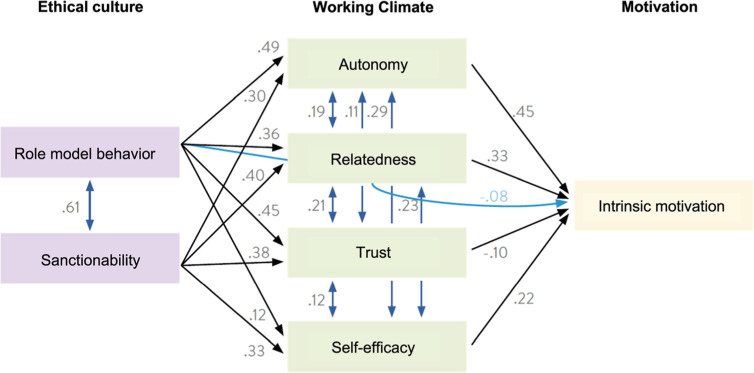
Structural equation model depicting relations between ethical culture, working climate, and motivation. Figure adapted from Hermanussen et al. (2022).

The theoretical model which was previously defined in Figure 1 was explored with SEM analysis using LISREL. We used a simplex model to specify relations between our variables and we calculated standardized regression coefficients. To reach an acceptable fit we added the effect of role model behavior on intrinsic motivation to the model. Figure 2 shows the eventual model including the standardized regression coefficients. Model fit was good, *χ*^2^ = .12, *df* = 3, *p* = .989, GFI = 1.00, AFGI = 1.00, and RMSEA <.001. All estimated effects as reported in Figure 2 are statistically significant. The strongest effect is reported between role model behavior and autonomy (.49; a medium-strong effect), indicating that an increase in role model behavior coincides with an increase in autonomy. The second strongest effect is reported between autonomy and intrinsic motivation (.45; a medium-strong effect), indicating that an increase in autonomy coincides with an increase of intrinsic motivation. The weakest effect can be found between role model behavior and intrinsic motivation (−.08; a weak effect), indicating that a decrease in role model behavior coincides with a decrease in intrinsic motivation. While this effect is small, it was significant, and it did improve overall fit of the model. The unexplained variance from this model on intrinsic motivation comes to 44%, which means that 56% of the variance is explained. There is no absolute threshold for an acceptable amount of explained variance (Fichman, 1999). In social sciences 60% explained variance (and sometimes even less) is generally accepted to be satisfactory (Hair et al., 2014).

The results confirm that nearly all variables influence one and another in the way we would expect based on our conceptual model (all variables are retained in the model and the reported effects are significant), although the strength of the relationships varies. The weak effects of trust and role model behavior on intrinsic motivation are exceptions.

## Discussion

This study has employed SEM analysis to investigate whether ethical culture impacts working culture variables and ultimately intrinsic motivation. We found that most of the anticipated relations specified in our theoretical model held true. Both ethical culture variables (i.e., role model behavior and sanctionability) were positively related to one and other and to the working climate variables (i.e., autonomy, relatedness, trust, and self-efficacy). The working climate variables were also positively associated to one and other and almost all of them were positively related to intrinsic motivation. However, there was a negative relation between role model behavior and intrinsic motivation and between trust and intrinsic motivation.

Our study thus suggests that role model behavior and sanctionability are important dials to be turned when it comes to influencing working climate variables and intrinsic motivation. This finding is in line with a wealth of research in the broader field of ethical culture and ethical leadership demonstrating that an increase of ethical leadership is associated with all sorts of positive outcomes (e.g., Ng & Feldman, 2015). Moreover, longitudinal studies (e.g., Huhtala et al., 2021) suggest the robustness of these effects. In the light of these findings the most obvious explanation for the weak negative relationship between role model behavior and intrinsic motivation seems to be that it is an anomaly, likely introduced by variables not captured in this study. However, a study by Bormann et al. (2018) looking into variability of ethical leadership provides an alternative explanation for the weak relationship between role model behavior and intrinsic motivation. Their study shows that high levels of variability (i.e., inconsistency) of ethical leadership is negatively associated with trust at the unit-level (i.e., a smaller part of an organization). Considering these results Bormann et al. (2018) suggest that overall impact of ethical leadership cannot be fully captured unless the unit-level is also considered. In other words, if ethical leadership is consistently applied across all units, differential effects may appear even while the overall impression of ethical leadership might be positive. The importance of approaching organizations at multiple levels is further enforced by Kangas et al. (2017), demonstrating that at an individual level perceptions of a strong ethical organizational culture were associated with less sickness absences, while this was not found at the unit-level.

Still another explanation might be found in needs frustration (Vansteenkiste & Ryan, 2013). Role model behavior might have frustrated feelings of competence by social comparison. Indeed, research (e.g., Boissicat et al., 2012; Boissicat et al., 2020) suggests that social comparison may impact feelings of competence, yet the relationship is complicated and not necessarily a negative one. For feelings of competence to be negatively affected, it seems that a person needs to identify with their target of comparison and that the comparison has to be a downward one (the target is perceived to be less competent). These studies were largely done with children (instead of adults) in a classroom setting (instead of the workplace), so without further research in this specific target audience and context and additional data on social comparison, it is hard to say if previous findings apply to our study.

Regarding trust and intrinsic motivation, it seems that only a limited number of studies have been conducted that connect the two concepts together. It seems that either trust and intrinsic motivation are positively related either directly or indirectly through moderation or mediation (e.g., Conchie, 2013). However, we have not encountered studies where the two were negatively (i.e., inversely) related. Possible explanations include the ones offered in the previous paragraph (i.e., an anomaly introduced by a variable that was not measured or differential effects across levels in the organization). However, a third possible explanation may come from a meta-review (i.e., a review based on a multitude of meta-analyses) of trust in the workplace (Dirks and de Jong, 2022). In their review Dirks and de Jong (2022) state that: ‘Despite multiple clarification efforts, the trust literature continues to be riddled with conceptual ambiguities and operational deficiencies’ (p. 265). In other words, even though thousands of studies have been conducted on the topic it continues to be difficult to agree on what exactly the construct of *trust* is, or how it should be measured. Considering the limited number of studies connecting intrinsic motivation and trust it is thus possible that our study and those studies employed different constructs.

### Limitations and Future Research

Some limitations apply to our research that could be addressed in future research. Firstly, while the study is cross-sectional in nature the data were not collected at the same time. This was due to practical constraints. The data collection of the larger Hermanussen et al. (2022) study, of which this study was a part, was confined to periods when audits were conducted. As these are predominantly conducted serially, we could thus only collect data when an audit ‘naturally’ occurred. In total, the collection of data in this study spanned over one and a half years. A period in which macro influences can change. One such example was the onset of the era of Covid-19. Some data were collected before and some data during the Covid-19 period. This period introduced such dramatic changes to the workplace, that it is likely variables in our study would have been impacted. Where possible, future research should seek out possibilities to collect the data all at the same time, of at least within a short period of time.

Secondly, the multilevel structure of our data may entail that effects may vary across layers within our SVET organizations. Participants in our study are part of a layer (sometimes sub-divided into teams) which are in turn part of an SVET organization. Our current analysis (SEM) does not capture differential effects on these layers. Multi-level structural equation modelling would have been able to do so, however for it to be reliable there need to be a certain number of participants at each layer (e.g., Maas & Hox, 2005). Due to the nature of some of the layers (e.g., members of the board rarely exceed three individuals) and the relatively limited amount of SVET organizations in our study, multi-level structural equation modelling was not applicable. However, future research could apply multi-level equation modelling if a larger sample of SVET organizations was obtained with a focus on layers that contain more participants (e.g., the staff layer). Such analysis might shed a light on the unexpected results in this study.

Thirdly, as per the design of this study (i.e., cross-sectional) we were only able to capture a snapshot of our variables in time. As such we were not able to measure whether the auditing approach had any effect on the long term. We recommend future research to employ pre-post- or repeated measures designs, to see if development-oriented auditing approaches can inspire soft-control meaningfully over time.

Fourthly, with larger sample sizes there is a risk of overfitting the model during SEM analysis. In this case the data might suffer from limited generalizability. Furthermore, what exactly constitutes a good fit has been an area of scientific dispute in which guidelines may vary. There is always some degree of subjectivity in determination of fit (Tomarken & Waller, 2005), due to this subjectivity there is a chance that a less-than-optimal model may have been fitted to the data.

Finally, we only used self-report measures which may suffer from a several reliability and validity issues caused by biases such as social-desirability bias (e.g., Fisher & Katz, 2000), while a large sample size may alleviate some of the validity and reliability issues it would have been better to use another data source not reliant on self-reports. We suggest that future research incorporate such a data source or multiple data sources so that triangulation of data will be possible and validity can be better ensured.

While this study has given insight into theoretical mechanisms underlying intrinsic motivation in the workplace it has not yielded ways to improving it; whereas we now which dials can be turned it remains somewhat unclear how to dial them. Three studies from other fields may help with this. Firstly, Ahmadi et al. (2023) present a classification of common behaviors teachers use to motivate their students. Secondly, Aelterman et al. (2018) demonstrate a circumplex model which presents a fine-grained analysis of teacher behaviors according to autonomy support, structure, chaos, and control. This study suggests that some motivational behaviors might be more motivating than others (i.e., some behaviors only enable need satisfaction, while others actively support it). Finally, some studies (e.g., Vansteenkiste et al., 2009) were able to cluster students into motivation types based on both quantity and quality of motivation. Where does this leave us? We have at our disposal, a set of motivating behaviors, a way to classify them so that they are need supporting, and a way to identify students that may profit from interventions the most. We suggest that future research uses all previous three to see if they also apply to an organizational context. Managers can select need-supporting behaviors that align with sanctionability and role model behavior and target them towards employees that may need them the most. In this way the current study is combined with insights from the broader field of motivation research.

### Practical Implications

In line with previous research, we suggest that organizations invest in guarding or improving the prevailing ethical culture as it appears to contribute to a sound working climate. Concretely, we advise those in management positions to exhibit the behavior they expect from their team members, but also to demonstrate that rules are upheld within the organization (i.e., rewarding positive behavior and sanctioning negative behavior). We suggest that role model behavior and sanctionability be applied in a uniform manner throughout the organization to avoid differential and possibly counteractive effects across the various layers in the organization.

## Conclusion

This study has demonstrated that ethical culture variables can impact working climate variables, which in turn can impact intrinsic motivation, in the context of an SVET development-oriented audit approach. This suggests that organizations can profit from investing in the prevailing ethical culture. However, future research is needed to establish whether such a development-oriented audit approach can contribute to improving an ethical culture and to discern the differential influences of the various layers involved in SVET organizations.

## Supplemental Material

Supplemental Material - What Lies Beneath: A Development-Oriented Auditing Approach to Understand Organisations Beyond the Surface of Hard-Control Supplemental Material for What Lies Beneath: A Development-Oriented Auditing Approach to Understand Organisations Beyond the Surface of Hard-Control by Jorrick Beckers, José Hermanussen, Ronald Stevens, and Ingrid van der Pluym in Psychological Reports

## Data Availability

The research data underlying this article are stored online at Open Science Framework (OSF) and accessible through https://doi.org/10.17605/OSF.IO/42Q3Z.
