# Supplementary material for: What Lies Beneath: A Development-Oriented Auditing Approach to Understand Organizations Beyond the Surface of Hard-Control
Source: Psychol Rep. 2024 Oct 13;129(4):3469–88. doi: 10.1177/00332941241283199 (PMC13287429; doi:10.1177/00332941241283199)
Supplement: Supplemental Material - What Lies Beneath: A Development-Oriented Auditing Approach to Understand Organisations Beyond the Surface of Hard-Control [file sj-pdf-1-prx-10.1177_00332941241283199.pdf]

Appendix 1

The full Soft-controls Questionnaire for the teaching staff layer. Questions have been translated from Dutch.

Sanctionability scale

Item #	Item
1	In my educational team, we hold members accountable if they harm educational quality
2	In my educational team, we highly appreciate being passionate about education
3	In my educational team, we take action towards members that harm educational quality
4	In my educational team, we are aware of behaviour that increases educational quality

Role model behavior scale

Item #	Item
1	My team manager propagates the importance of high-quality education convincingly
2	My team manager’s behaviour visibly improves a culture of accountability
3	My team manager’s behaviour is congruent with norms and values set by the board
4	My team manager honours agreements regarding educational quality within our institute
5	In my educational team it is common practice to have an open dialogue about professional standards between tea[illegible] team manager

Autonomy scale

Item #	Item
1	I feel I can do my work in any way I see fit
2	In my educational team there is room for what I find truly important
3	I feel my workload is fitting, not too comprehensive
4	My team manager guards that critical reflection about educational quality is a regular part of the educational team’s behaviour

Relatedness scale

Item #	Item
1	As an educational team we are often talking about core dilemmas in our workplace
2	I notice that the educational team holds a favourable attitude about me as a team member
3	I notice that the educational team members are really involved in each other’s work
4	I feel that the educational team members show a personal interest in me as a team member
5	The relation between the team manager and the educational team is strengthened by mutual involvement
6	I feel a bond with my fellow educational team members
7	I feel personally connected to my educational team

Trust scale

Item #	Item
1	There is an atmosphere of mutual trust between the educational team and the team manager
2	In my educational team everyone takes norms, values, and procedures seriously
3	In my educational team everyone one treats one and other with respect
4	In my educational team members trust the team manager

Self-efficacy scale

Item #	Item
1	I doubt about whether I can do things right in my educational team (inverted item)
2	I can complete complex tasks in our educational team
3	I feel adequately equipped to handle my role in the educational team
4	I am satisfied with the effect of my performance within the educational team

Intrinsic motivation scale

Item #	Item
1	I experience joy in my team
2	I find my work in the team relevant and meaningful
3	I find my work in the team challenging
4	I am enthusiastic about my current function in the team

Results

Confirmatory Factor Analysis

Factor Loadings

Factor	Indicator	Estimate	SE	Z	p
SANCTIONABILITY	SANC_1_item_25	0.880	0.0263	33.5	< .001
	SANC_2_item_26	0.523	0.0243	21.5	< .001
	SANC_4_item_30	0.848	0.0263	32.2	< .001
	VERT_5_item_28	0.521	0.0236	22.1	< .001
ROLE MODEL BEHAVIOR	item_31	0.833	0.0233	35.7	< .001
	item_32	0.842	0.0245	34.3	< .001
	item_33	0.725	0.0220	32.9	< .001
	item_34	0.748	0.0226	33.1	< .001
	SELF_4_item_17	0.780	0.0266	29.4	< .001
AUTONOMY	AUTO_1_item_6	0.489	0.0228	21.5	< .001
	AUTO_2_item_16	0.615	0.0240	25.6	< .001
	AUTO_3_item_18	0.432	0.0280	15.4	< .001
	AUTO_4_item_29	0.614	0.0283	21.7	< .001
RELATEDNESS	RELA_1_item_4	0.461	0.0314	14.7	< .001
	RELA_2_item_5	0.500	0.0234	21.4	< .001
	RELA_3_item_7	0.576	0.0207	27.9	< .001
	RELA_4_item_13	0.644	0.0262	24.5	< .001
	RELA_5_item_14	0.631	0.0242	26.1	< .001
	RELA_6_item_15	0.644	0.0283	22.8	< .001
	RELA_7_item_21	0.602	0.0219	27.4	< .001
TRUST	VERT_1_item_1	0.780	0.0257	30.3	< .001
	VERT_2_item_2	0.635	0.0261	24.4	< .001
	VERT_3_item_23	0.609	0.0268	22.7	< .001
	VERT_4_item_24	0.851	0.0246	34.6	< .001
SELF-EFFICACY	SELF_2_item_9_INVERTED	0.424	0.0318	13.3	< .001
	SELF_3_item_10	0.437	0.0228	19.1	< .001
	SELF_5_item_19	0.523	0.0232	22.6	< .001
	SELF_6_item_20	0.631	0.0219	28.9	< .001
INTRINSIC MOTIVATION	INTR_1_item_3	0.617	0.0212	29.1	< .001
	INTR_2_item_11	0.526	0.0188	28.0	< .001
	INTR_3_item_12	0.657	0.0222	29.6	< .001
	INTR_4_item_22	0.717	0.0219	32.8	< .001

Factor Estimates

Factor Covariances

		Estimate	SE	Z	p
SANCTIONABILITY	SANCTIONABILITY	1.000 [a]			
	ROLE MODEL BEHAVIOR	0.682	0.0201	33.9	< .001
	AUTONOMY	0.740	0.0262	28.3	< .001
	RELATEDNESS	0.655	0.0242	27.1	< .001
	TRUST	0.713	0.0214	33.4	< .001
	SELF-EFFICACY	0.491	0.0302	16.3	< .001
	INTRINSIC MOTIVATION	0.471	0.0286	16.5	< .001
ROLE MODEL BEHAVIOR	ROLE MODEL BEHAVIOR	1.000 [a]			
	AUTONOMY	0.838	0.0227	36.9	< .001
	RELATEDNESS	0.602	0.0245	24.5	< .001
	TRUST	0.815	0.0148	55.2	< .001
	SELF-EFFICACY	0.411	0.0303	13.6	< .001
	INTRINSIC MOTIVATION	0.482	0.0262	18.4	< .001
AUTONOMY	AUTONOMY	1.000 [a]			
	RELATEDNESS	0.883	0.0187	47.3	< .001
	TRUST	0.847	0.0207	40.9	< .001
	SELF-EFFICACY	0.830	0.0239	34.7	< .001
	INTRINSIC MOTIVATION	0.923	0.0170	54.2	< .001
RELATEDNESS	RELATEDNESS	1.000 [a]			
	TRUST	0.780	0.0197	39.5	< .001
	SELF-EFFICACY	0.677	0.0241	28.1	< .001
	INTRINSIC MOTIVATION	0.752	0.0188	40.0	< .001
TRUST	TRUST	1.000 [a]			
	SELF-EFFICACY	0.518	0.0292	17.7	< .001
	INTRINSIC MOTIVATION	0.536	0.0263	20.4	< .001
SELF-EFFICACY	SELF-EFFICACY	1.000 [a]			
	INTRINSIC MOTIVATION	0.768	0.0208	37.0	< .001
INTRINSIC MOTIVATION	INTRINSIC MOTIVATION	1.000 [a]			

[a] fixed parameter

Model Fit

Test for Exact Fit

χ^2	df	p
5255	443	< .001

Fit Measures

				RMSEA 90% CI			
CFI	TLI	SRMR	RMSEA	Lower	Upper	AIC	BIC
0.788	0.763	0.0900	0.0938	0.0916	0.0961	86690	87289

Path Diagram

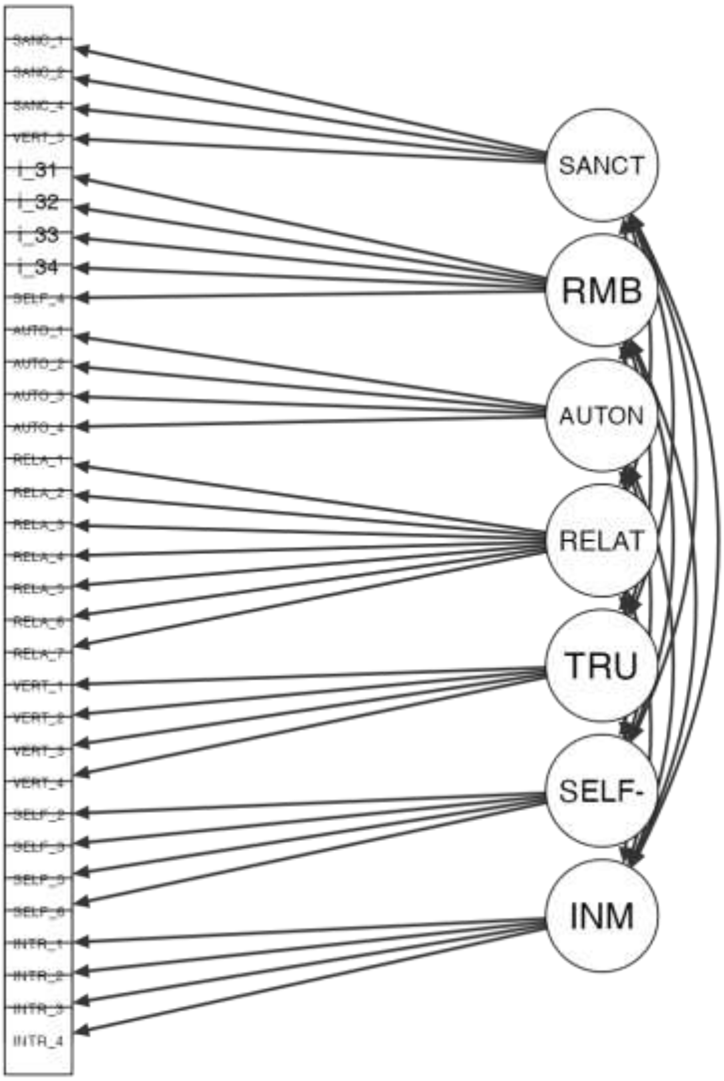

References

[1] The jamovi project (2022). *jamovi*. (Version 2.3) [Computer Software]. Retrieved from https://www.jamovi.org.

[2] R Core Team (2021). *R: A Language and environment for statistical computing*. (Version 4.1) [Computer software]. Retrieved from https://cran.r-project.org. (R packages retrieved from MRAN snapshot 2022-01-01).

[3] Rosseel, Y., et al. (2018). *lavaan: Latent Variable Analysis*. [R package]. Retrieved from https://cran.r-project.org/package=lavaan.

[4] Epskamp, S. (2017). *semPlot: Path Diagrams and Visual Analysis of Various SEM Packages' Output*. [R package]. Retrieved from https://cran.r-project.org/package=semPlot.
